# Purinergic Tuning of the Tripartite Neuromuscular Synapse

**DOI:** 10.1007/s12035-023-03317-8

**Published:** 2023-04-05

**Authors:** Carlos Sousa-Soares, José Bernardo Noronha-Matos, Paulo Correia-de-Sá

**Affiliations:** 1 Laboratório de Farmacologia e Neurobiologia, MedInUP, Instituto de Ciências Biomédicas Abel Salazar (ICBAS), Universidade do Porto, R. Jorge Viterbo Ferreira, 228, 4050-313 Porto, Portugal; 2 Centro de Investigação Farmacológica e Inovação Medicamentosa (MedInUP), Instituto de Ciências Biomédicas Abel Salazar (ICBAS), Universidade do Porto, Porto, Portugal

**Keywords:** Adenosine, Adenosine 5′-triphosphate, Neuromuscular junction, Perisynaptic Schwann cells, Purines

## Abstract

The vertebrate neuromuscular junction (NMJ) is a specialised chemical synapse involved in the transmission of bioelectric signals between a motor neuron and a skeletal muscle fiber, leading to muscle contraction. Typically, the NMJ is a tripartite synapse comprising (a) a presynaptic region represented by the motor nerve ending, (b) a postsynaptic skeletal motor endplate area, and (c) perisynaptic Schwann cells (PSCs) that shield the motor nerve terminal. Increasing evidence points towards the role of PSCs in the maintenance and control of neuromuscular integrity, transmission, and plasticity. Acetylcholine (ACh) is the main neurotransmitter at the vertebrate skeletal NMJ, and its role is fine-tuned by co-released purinergic neuromodulators, like adenosine 5′-triphosphate (ATP) and its metabolite adenosine (ADO). Adenine nucleotides modulate transmitter release and expression of postsynaptic ACh receptors at motor synapses via the activation of P2Y and P2X receptors. Endogenously generated ADO modulates ACh release by acting via co-localised inhibitory A_1_ and facilitatory A_2A_ receptors on motor nerve terminals, whose tonic activation depends on the neuronal firing pattern and their interplay with cholinergic receptors and neuropeptides. Thus, the concerted action of adenine nucleotides, ADO, and ACh/neuropeptide co-transmitters is paramount to adapting the neuromuscular transmission to the working load under pathological conditions, like *Myasthenia gravis*. Unravelling these functional complexities prompted us to review our knowledge about the way purines orchestrate neuromuscular transmission and plasticity in light of the tripartite synapse concept, emphasising the often-forgotten role of PSCs in this context.

## Introduction

The neuromuscular junction (NMJ) is a specialised chemical synapse involved in the transmission of electric signals between motor neurons and skeletal muscle fibres that are necessary for muscle contraction. The presynaptic region of the NMJ comprises the demyelinated part of the motoneuron axon terminal, which is normally shielded by perisynaptic Schwann cells (PSCs) [1, 2]. Unlike Schwann cells (SCs) that wrap the axons of myelinated motor neurons, PSCs are non-myelinating cells that play a fundamental role in the formation, maintenance, and regulation of skeletal NMJs [2, 3]. The synaptic cleft is a gap that separates the pre- and postsynaptic regions and contains a basal lamina of specialised extracellular matrix [1]. The postsynaptic motor endplate consists of deep plasma membrane invaginations (synaptic folds) and crests, where muscle-type α1 subunit-containing nicotinic receptors (nAChRs) are clustered. In vertebrates, the main neurotransmitter released by motor nerve terminals is acetylcholine (ACh), which accumulates only very briefly in the synaptic cleft during neuronal firing due to the presence of highly active acetylcholinesterase (AChE) enzymes. Activation of α1-containing nicotinic receptors by ACh released from motor nerve terminals triggers small depolarisations of the motor endplate region. Synchronisation of plasma membrane depolarisations results in the generation of an action potential, leading to subsequent skeletal muscle contraction [3].

Besides its role in muscle contraction, ACh regulates its release through the activation of presynaptic nAChRs and muscarinic receptors (mAChRs). In addition to facilitatory nAChRs containing α3β2 subunits [4, 5], nerve terminals are also endowed with M_1_ (facilitatory) and M_2_ (inhibitory) mAChRs [6, 7]. It has also been demonstrated that ACh spillover from the neuromuscular synapse participates in a negative feedback loop to restrain its release through the activation of nAChRs containing α7 subunits (α7 nAChRs) located on the plasma membrane of PSCs [8, 9]. Thus, increasing evidence suggests that PSCs can sense and tune neuromuscular transmission efficacy [10–13]. Besides ACh, other released signalling molecules (e.g. glutamate, purine nucleotides or nucleosides, and neuropeptides) play important roles in neuromuscular transmission regulation [14–16], namely by interfering with the activity of PSCs [8, 10–13]. In this review, we will briefly summarise the modulatory roles of adenosine (ADO) and adenine nucleotides on neuromuscular transmission; emphasis will be given to the role of distinct purinoceptor subtypes and their interplay with other neuromodulatory signalling pathways at both synaptic and perisynaptic levels. Understanding the crosstalk between cholinergic and purinergic cascades that regulate neurotransmitter release given the tripartite synapse concept may be relevant to guide the rationale of therapeutic approaches for diseases affecting the safety margin of neuromuscular transmission, such as myasthenia gravis and other myasthenic syndromes [17].

## Pre- and Postsynaptic Signalling by Adenine Nucleotides

### ATP is Co-released with ACh: Co-transmission and Neuromodulation by Purines

Considered the “molecular currency unit” found in all forms of life, adenosine 5′-triphosphate (ATP) is also known for its role as a neurotransmitter and neuromodulator both in central and peripheral synapses [18]. Unlike other nucleotides or their metabolites, ATP is stored in synaptic vesicles together with other neurotransmitters, such as ACh in cholinergic neurons. The ACh:ATP ratio in synaptic vesicles has been estimated to be between 1:1 to 10:1, depending on the technique and preparation used [19–22]. As such, ATP is co-released with ACh during neuromuscular transmission, and it accumulates at the synaptic cleft in a frequency-dependent manner during neuronal activity [16, 23, 24].

ATP can also originate from twitching skeletal muscle fibres in amphibian and rodent NMJs [25–28]. Pharmacological evidence suggests that pannexin-1 hemichannels mediate the release of ATP from skeletal myotubes [29]. Estimates about the proportion of ATP directly released from contracting muscle fibres vis a vis that of neuronal origin range between 15 and 80%; this contention depends on experimental settings concerning the use of low- vs high-frequency nerve stimulation patterns and the pre- vs post-junctional selectivity of the neuromuscular blockers used to paralyse skeletal nerve fibres [25–28].

Under appropriate conditions (e.g. hypotonicity, stimulation with uracil nucleotides), cultured SCs are also able to release ATP; yet, the relevance of this phenomenon in the context of PSC signalling and neuromuscular transmission remains to be elucidated in situ [30–32]. PSCs are also able to release ATP via a yet undisclosed mechanism that may be involved in the modulation of Ach release via the activation of presynaptic purinoceptors (see below) [10].

Neuromodulation by adenine nucleotides occurs either directly, via the activation of ionotropic P2X and metabotropic P2Y receptors (P2XRs and P2YRs, respectively) [18], or indirectly, after their extracellular breakdown into ADO and subsequent activation of P1 receptors, which include four distinct subtypes (A_1_, A_2A_, A_2B_, and A_3_; see below) [33]. Nerve terminals, skeletal muscle fibres, and PSCs express various subtypes of P2XRs and P2YRs (see Table 1) [34–36]. Yet, the pathophysiological relevance of ATP and its metabolite adenosine 5′-diphosphate (ADP) in the control of neuromuscular transmission is far from being consensual, mostly because research has been conducted under distinct experimental conditions, which include the use of different preparations from diverse animal species and developmental stages. Most often, no attempt has been made to separate P2- from P1-mediated effects [16].

**Table 1:** Pathophysiological role for P1 and P2 purinoceptors on neuromuscular transmission. Adenine nucleosides and nucleotides may exert other roles at the NMJ, but their targets and/or signalling mechanism still require pharmacological characterization

Receptor	Proposed Location	Proposed Function and Signaling	Organisms	Role in Neuromuscular Disorders	References
P2Y_12_	Nerve Terminals	Inhibition of ACh Release	Frog	Unexplored	[41]
P2Y_13_	Nerve Terminals	Inhibition of ACh Release	Mouse	Unexplored	[59, 169]
P2X7	Nerve Terminals	Facilitation of ACh release	Mouse	Unexplored	[48, 49, 55]
A_1_	Nerve Terminals	Inhibition of ACh release via inhibition of N-, and/or P/Q-type VGCCs	Rat, Mouse, Frog	Pharmacological inhibition of the receptor increases evoked ACh release in TIMG	[28, 38, 46, 78, 88]
	PSCs	Release of calcium from intracellular stores	Frog, Mouse	Unexplored	[11, 34, 104]
A_2A_	Nerve Terminals	Facilitation of spontaneous and/or evoked ACh release via the activation of quiescent L-type VGCCs	Rat, Mouse	Receptor activity compromised in ALS and *Myasthenia gravis* mouse and rat models, respectively. Pharmacological activation rescues neuromuscular transmission in myasthenic rats	[28, 69, 77, 102, 111, 170]
A_2B_	Nerve Terminals	Unknown	Mouse	Unexplored	[104]
	Skeletal Muscle Fibers	Increase the opening probability of postsynaptic nAChRs	Mouse	Unexplored	[104, 106]
A_3_	Nerve Terminals	Inhibition of ACh release via suppression of N-, L- or P/Q-type VGCCs	Mouse	Unexplored	[104, 108]
	Muscle Fibers	Unknown	Mouse	Unexplored	[104]

### P2Y Receptor-mediated Signalling

Attenuation of quantal ACh release by ATP may involve the activation of one or more types of pertussis-toxin-sensitive G_i/o_-coupled P2Y (most likely the P2Y_12_R and/or P2Y_13_R subtypes), but not ionotropic P2XRs, at the NMJ of amphibia [37, 38] and rodents [35]. Immunohistochemistry and pharmacological data point towards the involvement of the ADP-sensitive P2Y_13_R as the main purinoceptor mediating the inhibitory effect of ATP on ACh release at the mouse neuromuscular synapse. However, this is not consensual given to the fact that ATP-induced decreases in non-quantal transmitter release at mammalian NMJs may also involve other P2YR subtypes coupled to different intracellular signalling pathways [39, 40]. Data in amphibia considerably differ from that obtained in mammals. The mechanism underlying ATP-mediated inhibition of ACh release in frogs involves ADP-sensitive P2Y_12_Rs, but not P2Y_13_Rs [41], via an intracellular mechanism linking the production of reactive oxygen species (ROS; including H_2_O_2_), oxidation of the synaptosomal-associated protein 25 (SNAP25), and decrease in synaptic vesicle release probability [42, 43]. The coupling of inhibitory P2YRs to a second ROS-independent signalling pathway involving PLC/PKC/PLA_2_/COX activation has also been proven [37, 42, 44]. In support of this theory, byproducts of PLA_2_ and COX activity, like arachidonic acid and prostaglandin E_2_, are known to decrease ACh release by suppressing Ca^2+^ influx into frog motor nerve endings [45].

It is also worth noting that modulation of evoked transmitter release by ATP is more often observed under low neuronal stimulation frequencies (≤ 1 Hz). It remains to be elucidated if the inhibitory role of ATP is maintained under more physiological nerve-firing conditions (e.g. 50–100 Hz bursts), which are known to affect neuromodulation by other purines, such as ADO. Previous studies from our group using selective receptor agonists and antagonists showed that ADO (and its analogues) could activate both presynaptic A_1_ inhibitory/A_2A_ facilitatory receptors depending on the motor nerve stimulation paradigm and, thus, on the amount of ADO accumulated at the rat NMJ (see below) [6, 46]. Contrariwise, the P2 purinoceptor(s) involved in the aforementioned ATP-mediated effects require comprehensive investigations.

### P2X Receptor-mediated Signalling

Besides the predominant inhibitory effect of P2YRs at the NMJ, the net effect of ATP may be balanced via the activation of Ca^2+^-permeable facilitatory P2XRs [47–49]. Yet, independent studies have shown that non-hydrolysable ATP analogues can either facilitate or inhibit ACh release from motor nerve terminals [38, 50, 51]. ATP significantly decreases the amplitude of nerve-evoked muscle twitches of rat extensor digitorum longus (EDL) and soleus muscles while increasing the twitch amplitude at the mouse EDL, diaphragm, and soleus muscles [52–54]. These findings confirm our contention that the effect of ATP on neuromuscular transmission significantly differs among species [52]. It is also interesting to note that the facilitation of ACh release by P2 receptors is normally coupled to the activation of presynaptic facilitatory nAChRs [50].

The presence of the P2X7R was detected on mammalian motor nerve terminals by immunofluorescence and electron microscopy, where it acts to increase synaptic vesicle exocytosis [48, 49, 55]. The P2X7R exhibits low desensitisation probability and high conductance to monovalent and divalent cations, like Ca^2+^, which might explain its ability to increase ACh exocytosis [33, 56, 57]. Nevertheless, the true relevance of P2X7R activation on neuromuscular transmission is still a matter of debate because some authors failed to reproduce the facilitation of transmitter exocytosis using P2X7R agonists in mammals and amphibia NMJs [37]. Downstream activation of pannexin-1 hemichannels may partially occlude the facilitatory effect of the P2X7R given that the formation of large membrane pores may allow the release of inhibitory adenine nucleotides among other substances with molecular weight up to 900 Da [55, 58]. The low affinity of the P2X7R for ATP suggests that it might have negligible activity under physiological conditions, even considering that this receptor is sensitised by transient decreases in extracellular Ca^2+^ levels that are normally observed during high-frequency stimulation trains [33]. Thus, one may speculate that activation of P2X7Rs on mammalian motor nerve terminals might only occur under pathological conditions (e.g. ischaemia or muscle damage) when extracellular ATP levels increase considerably [49].

## Extracellular Catabolism of Adenine Nucleotides and ADO Formation at the NMJ

ATP is sequentially hydrolysed into ADP, adenosine 5′- monophosphate (AMP), and ADO by extracellular ectonucleotidases at mammalian NMJs [24, 26, 59, 60]. However, the characterisation of the enzymes responsible for the extracellular ATP breakdown at neuromuscular synapses is still lacking. It has been demonstrated that nucleoside triphosphate diphosphohydrolase 2 (NTPDase2 or ATPase) is located in the vicinity of mammalian motor endplates [61], which may contribute to the preferential accumulation of ADP compared to that of AMP when exogenous ATP is used as substrate [24]. So, ATP and/or ADP may transiently accumulate at the NMJ during high-frequency neuronal firing or under pathological conditions. High extracellular ATP and/or ADP levels feed-forwardly inhibit AMP dephosphorylation to ADO by ecto-5′-nucleotidase/CD73, thus partially preventing its formation [46, 62]. Under such conditions (e.g. continuous stimulation with 50 Hz trains), neuromodulation through ATP/ADP-sensitive P2-mediated purinoceptors is likely to prevail over ADO-mediated signals operated by P1 receptors.

The rat NMJ is also equipped with an ecto‐AMP deaminase pathway that metabolises adenine nucleotides to inosine 5′-monophosphate (IMP), bypassing ADO formation [24]. Thus, while the ecto‐5′‐nucleotidase/CD73 pathway modulates the rate of ADO formation from released nucleotides, alternative AMP deamination to IMP controls the amount of AMP available for ADO formation. In the innervated frog sartorius muscle, extracellular endogenous amounts of ADO are also balanced by exo-AMP deaminase and ecto-5'-nucleotidase activity. When only one of these enzymes is inhibited, the evoked release of adenine nucleotides becomes undetectable. Data strongly suggest that each of these enzymes is able, on its own, to break down whole AMP resulting from the catabolism of released adenine nucleotides upon stimulation [25].

## ADO: Modulator of Neuromodulators at the NMJ

### ADO Formation and Inactivation at the NMJ

ADO is a purine nucleoside ubiquitously found in most synapses, where it exerts neuromodulatory roles via the activation of P1 receptors. Unlike neurotransmitters, ADO is not stored in synaptic vesicles nor released by exocytosis [18, 63]. The nucleoside is released as such from intracellular compartments via equilibrative nucleoside transporters (ENTs); ADO may also originate from the extracellular catabolism of ATP co-released with other neurotransmitters, including ACh (see above) [63, 64]. Intracellularly there are two major pathways leading to ADO formation: AMP hydrolysis catalysed by the cytosolic 5′-nucleotidase or hydrolysis of *S*-(5′-adenosyl)-L-homocysteine (SAH) by SAH hydrolase. Intracellular ADO accumulation is balanced by its formation and/or uptake from the extracellular milieu (via ENTs) and the activity of two intracellular enzymes, adenosine kinase (ADK, *K*_*m*_ for ADO in the nanomolar range) and adenosine deaminase (ADA, *K*_*m*_ for ADO in the micromolar range), converting the nucleoside into AMP and inosine, respectively [63, 65]. Since the *K*_*m*_ value of ADA was found to be at least ninefold higher than that of ADK, the preferential phosphorylation of ADO to AMP (a less membrane-permeable compound) constitutes the main driving force to take up ADO from the extracellular milieu while keeping low cytosolic levels of the nucleoside, both in peripheral and central nervous system synapses [66].

Contracting muscle fibres were also identified as a major source of ADO at several NMJs [22, 26, 28, 60]. ADO progressively accumulates when ecto-5′-nucleotidase/CD73 and ecto-ADA are inhibited in the innervated frog sartorius, thus suggesting that the nucleoside is released as such even in the absence of nerve stimulation. Using the muscle-type nAChR antagonist, *d*-tubocurarine, authors estimated that extracellular ADO amounts originated from 0.2 Hz twitching skeletal muscle fibres were roughly identical to the nucleoside released from stimulated motor nerve terminals [25]. These findings challenged a previously published paper showing that ADO release was essentially abolished in the presence of *d*-tubocurarine, providing that ecto-5′-nucleotidase/CD73 was also inhibited, hence suggesting that contracting muscle fibres contribute to most extracellular ADO released as such [22]. A major limitation of both studies’ estimations relies on the fact that *d-*tubocurarine abrogates the ACh/ATP release facilitatory drive due to the activation of α3β2-containing nicotinic autoreceptors on mammalian motor nerve terminals besides its ability to block α1-containing nAChRs on skeletal muscle fibres [4, 14, 67].

Using an HPLC technique, we showed that selective muscle paralysis with μ‐conotoxin GIIIB, a toxin that blocks muscle‐specific voltage‐gated Na^+^ channels without affecting neuronal function [4, 68], significantly (> 90%) decreased nerve‐evoked ADO outflow without much affecting (∼ 15%) the release of ATP (and related nucleotides) [28, 69]. Data also showed that endogenous ADO generated during high-frequency nerve stimulation of motor endplates of *myasthenia gravis* patients was insufficient to maintain transmitter release demand through activation of the A_2A_ receptor (A_2A_R)-mediated presynaptic facilitatory drive [28]. Interestingly, myasthenic patients have impaired oxidative metabolism and a noticeable shift to glycolytic metabolism in skeletal muscles during exercise, which yields to higher‐end Pi/ATP ratio and reduced levels of synaptic ADO levels [70]. Taken together, these findings strengthen the idea that deficient ADO release from myasthenic skeletal muscle fibres may contribute to explaining the neuromuscular deficits observed in patients, which cannot be compensated by the smaller ADO amounts formed from released adenine nucleotides. Curiously, both neuromuscular impairments and ADO neuromodulation deficits in myasthenic motor endplates could be rehabilitated using the nucleoside precursor, AMP, or methylprednisolone; the latter is a synthetic glucocorticoid immunosuppressant that is widely used to prevent myasthenic crisis because it also amplifies neuromuscular transmission by increasing endogenous ADO availability and activation of facilitatory A_2A_Rs, which promotes synaptic vesicle recycling and release [51, 69, 70].

More recently, increasing evidence has strengthened the idea that PSCs, along with motor nerve terminals and skeletal muscle fibres, are also able to release ADO [8, 10, 71–73]. We provided recent data suggesting that activation of α7 nAChRs on PSCs controls ACh spillover from motor endplates by promoting ADO outflow via ENT1 and retrograde activation of presynaptic inhibitory A_1_ (A_1_Rs) [8]. However, the mechanism(s) underlying ADO release from PSCs still need to be elucidated in the future (see discussion below).

Extracellular ADO accumulation is also fine-tuning regulated by the nucleoside inactivation mechanisms, both cellular uptake and deamination [60, 74, 75]. The presence of ecto-ADA at both mammalian and amphibian NMJs is responsible for the conversion of ADO into inosine in the synaptic cleft [24, 28, 60]. Motor synapses are also endowed with the ENT1 [75, 76]. Both uptake and deamination coordinate to remove extracellular ADO, thus contributing to fine-tuning regulate activation of P1 receptor subtypes on motor nerve terminals [46, 75, 77, 78].

### ADO-mediated Signalling via P1 Purinoceptors

Neuromodulation by ADO depends on the activation of metabotropic P1 receptors, which are sub-classified in A_1_, A_2A_, A_2B_, and A_3_ (Table 1). In rodents, A_1_R and A_2A_R display high affinity for ADO, while A_2B_R and A_3_R are low-affinity receptors. Most commonly, A_1_R and A_3_Rs couple to *G*_i/o_ proteins, while A_2A_R and A_2B_R are preferentially coupled to *G*_s_ protein subunits. As such, activation of A_2A_R and A_2B_R normally results in stimulation of adenylyl cyclase (AC), resulting in increases in intracellular 3′-5′-cyclic adenosine monophosphate (cAMP) and subsequent protein kinase A (PKA) activation. Conversely, activation of A_1_R and A_3_R subtypes results in the suppression of AC activity, thus decreasing cAMP levels and PKA activation [79]. It is noteworthy that these receptors may also couple to non-canonical pathways, thus regulating intracellular concentrations of Ca^2+^ and/or K^+^ through the activation of *G*_q_ protein-coupled mechanisms; others include a direct interference with ion currents across the plasma membrane via the βγ subunit of G proteins [63, 80], although some controversy still exists on this matter.

### A_1 _Receptors

**Fig. 1:**
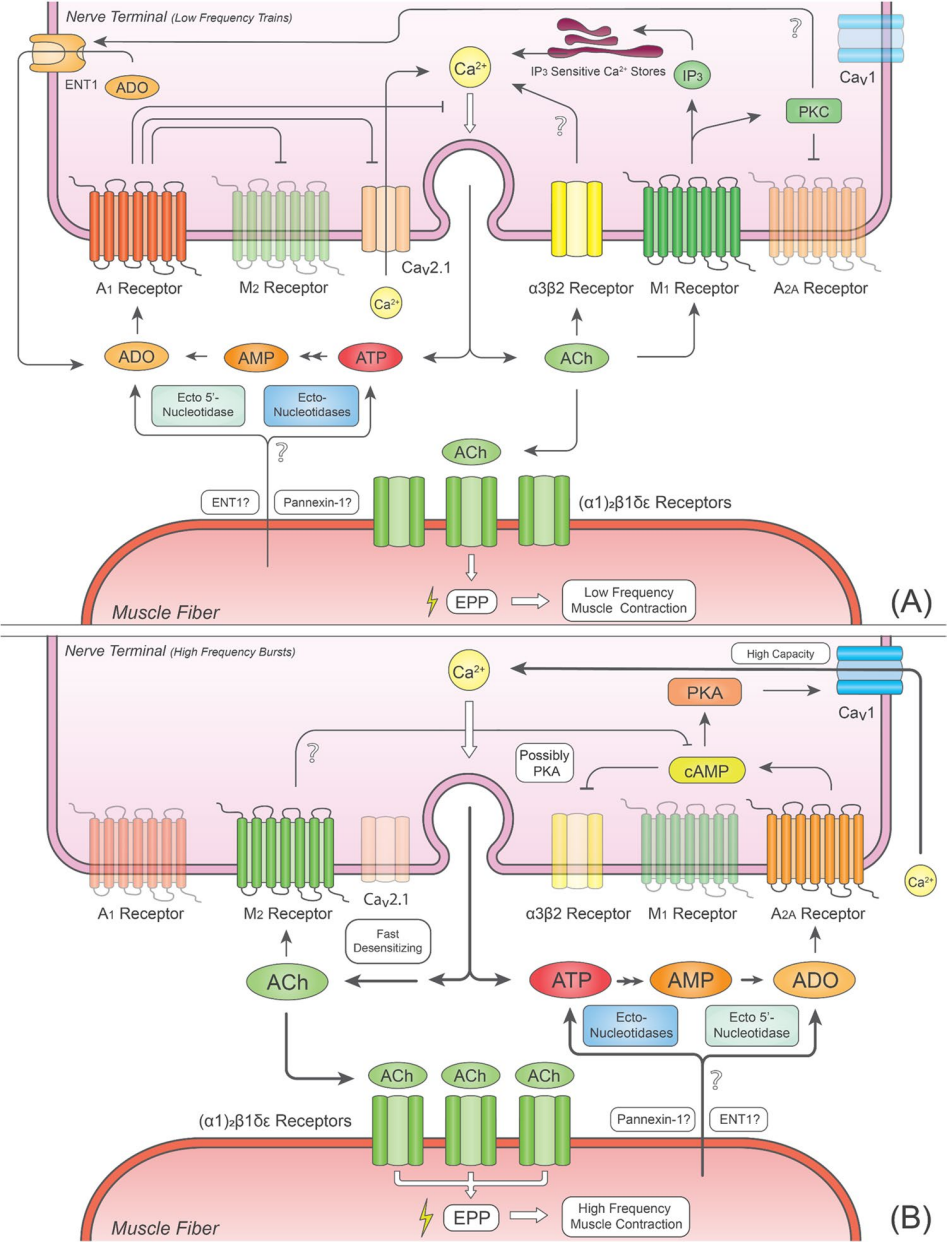
Crosstalk between purinergic and cholinergic auto-receptors to control nerve-evoked ACh release at the mammalian neuromuscular junction **A** under resting conditions and **B** during intense neuronal activity. Bold arrows indicate an increase in the strength of the proposed pathways. Predominant receptors operating in each condition appear as fully opaque, while non-predominant receptors appear as semi-transparent. See text for details. Abbreviations: ACh, acetylcholine; ADO, adenosine; AMP, adenosine 5′-monophosphate; ATP, adenosine 5′-triphosphate; cAMP, 3′-5′-cyclic adenosine monophosphate; ENT1, equilibrative nucleoside transporter 1; EPP, endplate potential; IP_3_, inositol 1,4,5-triphosphate; PKA, protein kinase A; PKC, protein kinase C

ADO released as such or formed from the extracellular breakdown of released ATP reaches very small amounts in non-stimulated motor endplates. ADO exerts a predominantly inhibitory effect on the release of ACh via A_1_R activation [46, 75, 78] when motor nerves fire at low activation frequencies [38, 74, 81]. The mechanism by which the A_1_R inhibits ACh release from motor nerve terminals is still poorly understood, but it is thought to involve direct inhibition of the secretory apparatus or inhibition of Ca^2+^ currents. At the frog NMJ, activation of A_1_R inhibits ACh release through a Ca^2+^-independent mechanism, suggesting a direct interference with proteins involved in vesicular exocytosis [82–84]. At mammalian NMJs, the A_1_R-mediated inhibition of ACh release depends on the inhibition of Ca^2+^ currents via N (Ca_v_2.2) [85] and P/Q (Ca_v_2.1) voltage-sensitive channels [86–88]. Since inhibition of N-type (Ca_v_2.2) Ca^2+^ currents is insufficient to affect both spontaneous and evoked ACh release from mammalian NMJ under resting conditions [86, 87, 89], reduction of transmitter release by A_1_Rs may predominantly rely on the suppression of Ca^2+^ influx via P/Q-type (Ca_v_2.1) channels (Fig. 1a), even though cross-talk between A_1_Rs and proteins of the exocytosis apparatus (such as Rab3A) via G protein βγ subunits has also been reported [90, 91].

The A_1_R located on motor nerve terminals may also be the effector of synaptic depression caused by other inhibitory signalling molecules, like the non-proteinogenic amino acid L-citrulline [72, 76]. This byproduct of nitric oxide (NO) resulting from the breakdown of L-arginine by nitric oxide synthase (NOS) [92, 93] inhibits nerve-evoked ACh release by increasing ADO outflow at the neuromuscular synapse via ENT1 transporters, fostering activation of the inhibitory A_1_R. In contrast to that observed with L-citrulline, ADO does not seem to play a role in NO-induced inhibition of ACh release from motor nerve terminals [72, 76].

### A_2A _Receptors

In 1991, previous results from Correia-de-Sá et al. demonstrated by electrophysiological and neurochemical methods that ADO A_1_-inhibitory and A_2_-excitatory receptors coexist at rat phrenic nerve terminals to fine-tune regulate evoked ACh release [78]. This seminal paper unravelled for the first time the neuronal activity of excitatory A_2A_Rs outside basal ganglia in the CNS.

Activation of A_2A_Rs at the mammalian NMJ is revealed when motor neurons fire at high-frequency rates and/or when nerve terminals are focally depolarised [46]. Under such conditions that roughly mimic the firing rate of motor neurons during initiation of normal respiratory or voluntary motion, ATP co-released with ACh reaches levels that are beyond the critical level required for ADO formation [19, 94]. Experimental data from our group showed that bypass of the initial feedforward inhibition of ecto-5′-nucleotidase/CD73 transiently caused by high ATP levels is required before ADO boosts at the motor endplate to allow subsequent activation of facilitatory A_2A_Rs [16, 46, 75].

The opposing effects of ADO at the mammalian NMJ may not be exclusively dependent on the nerve stimulation paradigm nor on the nucleoside concentration in the synaptic cleft, but it is also determined by its source. While ADO activates both A_1_R and A_2A_R depending on the extracellular concentration of the nucleoside, ADO resulting from the extracellular catabolism of ATP co-released with ACh preferentially activates facilitatory A_2A_Rs [46, 75, 94]. This feature, which is commonly observed in other peripheral and central synapses [95–98], may be attributable to the proximity of ecto-5′-nucleotidase/CD73 and A_2A_Rs, thus favouring the activation of this receptor subtype following ADO formation by AMP breakdown [95, 97].

The A_2A_R-mediated facilitation of ACh release from motor nerve endings is thought to be mainly caused by normally “quiescent” high-capacity L-type (Ca_v_1) VGCCs, which allow the influx of Ca^2+^ when “predominant” P/Q (Ca_v_2.1) channels are desensitised, such as during high-frequency neuronal firing (Fig. 1b) [28, 77, 86]. The mechanism downstream activation of the A_2A_R on motor nerve terminals is dependent on cAMP formation by AC [99, 100], leading to stimulation of PKA activity and subsequent Ca^2+^ influx through “quiescent” L-type (Ca_v_1) VGCCs (Fig. 1b) [101, 102]. Involvement of Ca^2+^ release from intracellular stores may also concur to facilitate ACh release from motor nerve terminals during intense neuronal activation [86, 102]. Phosphorylation of synapsin I by PKA may account to facilitate the mobilisation of synaptic vesicles to the active release zones following A_2A_R activation [51, 103], but this mechanism has never been directly addressed at vertebrate NMJs.

### A_2B _and A_3_ Receptors

Scarce evidence shows the presence and function of A_2B_R and A_3_R at the NMJ. Localisation of A_2B_R in presynaptic nerve terminals, as well as at the motor endplate region, has been observed at the mouse NMJ, but their roles are largely undefined [104]. Using high-resolution confocal microscopy, these authors also identified the A_1_R in PSCs and nerve terminals and the A_2A_R in the postsynaptic skeletal muscle, as well as in axon nerve terminals of the mouse NMJ. Despite this, the AC/cAMP/PKA pathway may be involved in the trafficking and clustering of nAChRs on the postsynaptic membrane, meaning that postjunctional A_2B_Rs are important for neuromuscular transmission efficacy and the long-term stability of the NMJ [105]. In the mouse diaphragm, activation A_2B_Rs with either adenosine or a synthetic ADO analogue increases the opening probability of nAChRs on skeletal muscle fibres. This effect was abolished in the presence of an AC inhibitor, thus confirming the involvement of the AC/cAMP/PKA pathway [106]. The PKA-mediated phosphorylation of the skeletal-type nAChR favours its opening probability and duration [107], prompting a putative synergistic interplay between postsynaptic A_2B_Rs and nAChRs on neuromuscular transmission, besides the role of this low-affinity ADO receptor subtype in NMJ stability promotion. The physiological significance of the A_2B_R activation on neuromuscular transmission remains to be elucidated given the fact that it requires high extracellular ADO amounts, which are normally unavailable at this particular synapse unless cellular damage due to ischaemia or inflammation is also present.

A_3_Rs have also been localised at presynaptic nerve terminals (and possibly the postsynaptic membrane) of mammalian NMJs. Activation of the A_3_R downregulates both spontaneous and evoked ACh release [104, 108]. This putative inhibitory effect on neuromuscular transmission may, indeed, be mediated by inosine, the ADO metabolite resulting from ADA activity [109]. Inosine decreases the frequency of MEPPs as well as the amplitude of nerve-evoked endplate potentials (EPPs) in mammalian but not in frog NMJs [74, 104, 108]. Inosine may decrease the release probability either directly, by activating low-affinity inhibitory A_3_Rs, or indirectly, through inhibition of ADO cellular uptake by ENTs [104, 108].

### Pathophysiological Implications of P1 Receptor Activation Deficits

The loss of function of A_2A_R seems to be a hallmark of ageing and some neuromuscular disorders (Table 1). While tonic activation of inhibitory A_1_R is largely preserved, deficits in the activity of excitatory A_2A_R were observed in diaphragm motor endplates of aged rats, thus contributing to the characteristic age-related neuromuscular transmission impairment [110]. Loss of the A_2A_R tone may also play a role in neuromuscular diseases, like myasthenia gravis and amyotrophic lateral sclerosis (ALS; see discussion below) [28, 111]. However, changes in the protein density of A_2A_R predominantly located on nerve axon terminals do not account for the functional deficits observed in the autoimmune myasthenia gravis (EAMG) rat model compared with naïve animals, as demonstrated by immunofluorescence confocal microscopy [69]. Myasthenia gravis is a B-cell-mediated and T-cell-dependent autoimmune disease characterised by the production of antibodies directed against muscle-type nAChRs containing α1 subunits; this attack reduces the number of effective nAChRs at motor endplates to nearly one-third of the normal amount, at least partially explaining the chronic muscle weakness that is characteristic of this disease [112]. Using rats with toxin-induced myasthenia gravis (TIMG), our group demonstrated that the A_2A_R-mediated facilitation of ACh release during high-frequency stimulation bursts is significantly impaired due to insufficient ADO outflow from myasthenic skeletal muscle fibres. Consequently, the necessary shift from fast desensitising P/Q (Ca_v_2.1) to facilitatory high-conductance L-type (Ca_v_1) VGCCs is compromised, which contributes to tetanic failure and reduced vesicular exocytosis [28]. Likewise, the endogenous levels of ADO were below those required to activate A_2A_R at both the *T*_Reg_/*T*_Helper_ immunological and the neuromuscular synapse in the EAMG rat model [69]. As such, this animal model presents deficits in sustaining muscle contraction during high-frequency neuronal firing (tetanic failure) and exaggerated T- and B-cell-mediated immune responses, leading to unrestrained production of antibodies against the muscle-type nAChR.

A strategy to experimentally overcome both immunological and neuromuscular transmission deficits in myasthenic animals was the rehabilitation of the A_2A_R tonus through in vitro application of AMP, which is rapidly converted into ADO by ecto-5′-nucleotidase/CD73 [28, 69]. Whether this strategy works in vivo remains to be elucidated, for instance using newly developed A_2A_R agonists (prodrugs) consisting of 2-substituted AMP derivatives that are locally activated by ecto-5′-nucleotidase/CD73 [113]. Selective activation of *A*_2A_Rs using CGS 21680C also specifically reduced the production of antibodies against the muscle-type α1-containing nAChRs by B-cells and increased the proliferation of *T*_reg_ cells in EAMG rats. Intraperitoneal treatment of these rats with CGS 21680C also decreased the myasthenic symptoms [114].

Besides the immunosuppressive effect, rehabilitation of the facilitatory A_2A_R-mediated tonus may also afford an explanation for glucocorticoid improvements of the neuromuscular transmission deficits in myasthenic patients. At the rat NMJ, methylprednisolone facilitates neurotransmitter release during high-frequency bursts; the effect was potentiated by blocking inhibitory muscarinic M_2_ and A_1_Rs, but it was prevented by blockage of facilitatory M_1_ and A_2A_Rs [51, 101]. Methylprednisolone favours ATP release under resting conditions, thus fostering ADO formation from ATP breakdown and, thereby, A_2A_R activation, allowing these receptors to play a facilitatory role from the beginning of nerve stimulation onwards. Phosphorylation of synapsin I clustered with vesicles in the reserve pool causes the priming of mature vesicles to the readily releasable pool, allowing maintenance of transmitter exocytosis during high-frequency stimulation [103, 115]. Taking this into consideration, together with the fact that activation of M_1_ and A_2A_Rs cooperate downstream to activate PKA, one may speculate that methylprednisolone facilitates ACh release by increasing the readily releasable pool during high-frequency stimuli through the prevention of vesicle clustering in the reserve pool [51, 115].

The putative involvement of adenosinergic signalling in the pathophysiology of ALS has also been reported [116]. ALS is a neurodegenerative disease leading to motor neuron dysfunction, resulting in impairment of neuromuscular transmission [117]. Using electrophysiology, Nascimento et al. demonstrated the role of A_2A_R on neuromuscular transmission in the ALS SOD1(G93A) mouse model, as well as its nuances during disease progression [111]. In ALS pre-symptomatic phase (4–6 weeks old mice), the A_2A_R-mediated excitation of the neuromuscular transmission had a higher magnitude than that found in age-matched controls, as demonstrated by increases in the mean amplitude and quantal content of EPPs, as well as in the frequency of MEPPs and appearance of giant MEPPs. Contrariwise, the CGS 21,680-induced A_2A_R-mediated facilitation was absent from symptomatic SOD1(G93A) mice (12–14 weeks old). In addition, the A_2A_R is overexpressed in lymphocytes from ALS patients, leading to intracellular cAMP accumulation in these cells [118]. This may indicate a putative role of A_2A_R in immunosuppression observed in ALS patients.

Regarding the controversies associated with the expression and function of A_3_R and A_2B_R at the NMJ, further investigations are mandatory to elucidate the role of these low-affinity ADO receptors on neurotransmitter release using animal models of skeletal muscle ischaemia/reperfusion, congenital and toxicological myopathies, and/or autoimmune and inflammatory insults where extracellular ADO (and inosine) levels may increase dramatically.

## ADO Modulates Cholinergic and Peptidergic Signalling at the NMJ

### Modulation of Cholinergic Autoreceptors Function

In addition to direct modulation of the neuromuscular transmission, evidence points towards crosstalk between co-localised P1 receptors, mAChRs and nAChRs at the NMJ [6, 73, 119, 120]. Motor nerve terminals are endowed with facilitatory M_1_ and inhibitory M_2_ muscarinic receptors (M_1_Rs and M_2_Rs, respectively), which coordinate their actions to adjust ACh release to neuronal activity [6, 119, 120]. The M_1_R positive feedback loop predominates during low-frequency neuronal firing, but it fades out upon increasing the neuronal firing rate. The opposite occurs concerning the M_2_R-mediated inhibition of ACh release (Fig. 1a and b) [6]. The muscarinic M_1_/M_2_ activation balance may also depend on the duration of neuronal activation [7]. Immunofluorescence staining of the mouse levator auris longus showed that M_1_Rs are absent from the motor endplate region but can be detected at presynaptic sites, possibly on the nerve terminal or PSCs [120].

Besides acting on mAChRs, ACh facilitates its release through the activation of fast desensitising nAChRs located on motor nerve terminals of both mice and rats [5, 121, 122]. Nicotinic facilitation of ACh release from motor nerve terminals of the rat phrenic nerve exhibits a pharmacological profile suggesting the involvement of α3β2 subunits-containing nAChRs (Fig. 1a) [4, 123]. The mechanism(s) underlying the facilitation of ACh release by presynaptic M_1_Rs and α3β2-containing nAChRs is still elusive. Experimental data suggest that release facilitation by M_1_Rs relies mainly on intracellular Ca^2+^ recruitment from inositol 1,4,5-triphosphate (IP_3_)-sensitive stores (Fig. 1a), being relatively insensitive to blockage of PKC activity [101, 124–126]. Most likely, α3β2-containing nAChRs may facilitate ACh release either through direct Ca^2+^ influx via the nicotinic pore or through indirect focal depolarisation of the nerve terminal membrane [4].

Increasing evidence suggests that cholinergic facilitation of ACh release is fine-tuning modulated by endogenous ADO generated at the neuromuscular synapse to adapt the neuromuscular transmission to the neuronal firing rate. Low endogenous levels of the nucleoside are generated when the neuronal firing pace is slow, which favours activation of inhibitory A_1_Rs, thus keeping the facilitation of neurotransmitter release by muscarinic M_1_ and α3β2 nAChRs fully operative to reduce failures of skeletal muscle contraction in response to nerve activity [4, 123]. Upon increasing the motoneuronal firing rate, the endogenous formation of ADO from released adenine nucleotides dramatically increases fostering the activation of facilitatory A_2A_Rs [46]. This shift from A_1_R to A_2A_R signalling contributes to attenuated facilitation of ACh release by M_1_Rs and α3β2 nAChRs, thus favouring the inhibitory control of the neuromuscular transmission handled by muscarinic M_2_Rs [6, 46, 127]. Tonic activation of A_2A_Rs suppresses α3β2 nAChR-mediated facilitation of ACh by a mechanism dependent on cAMP generation (Fig. 1b) [121, 123] and PKA activation [99, 101], which accelerates the desensitisation of nAChRs [128]. Moreover, the adaptive shift also triggers the recruitment of normally quiescent L-type (Ca_v_1) VGCCs, which might compensate for the loss of fast desensitising P/Q-type (Ca_v_2.1) currents to attenuate/prevent tetanic-induced synaptic depression during high-frequency neuronal activity (Fig. 1a and b; see above) [77, 86].

The mechanism by which M_2_Rs inhibits evoked ACh release remains largely unexplored but might involve a reduction in cAMP levels due to G_i/o_ protein coupling [125, 129] or to the modulation of a presynaptic effector mechanism downstream of Ca^2+^ entry, like that occurring with presynaptic A_1_Rs [127]. Neurochemical and electrophysiological data show that activation of A_1_ and M_2_ inhibitory receptors is mutually exclusive [6, 73, 127]. Besides preventing M_2_R-mediated inhibition of ACh release, full operation of the M_1_R also attenuates the A_2A_R-mediated facilitation through a mechanism involving PKC-induced phosphorylation of AC and/or its downstream intracellular signalling cascade (Fig. 1a) [101, 125].

### Modulation of Peptidergic Neurotransmission

A_1_R and A_2A_R play a pivotal role in the facilitation of nerve-evoked ACh release by neuropeptides at neuromuscular synapses [14, 130–132]. Calcitonin gene-related peptide (CGRP) released from large dense-core vesicles of mammalian motor nerve terminals [133, 134] facilitates ACh release by a mechanism involving stimulation of the AC activity [133, 135, 136], providing that A_2A_R are synchronously activated by endogenously generated ADO [14]. The mechanism underlying the synergism between A_2A_ and CGRP receptors to facilitate nerve-evoked ACh release is similar to that occurring between the purinergic receptor and forskolin, a direct AC activator [100], probably involving the coupling and/or recruitment of *G*_s_ proteins before activation of the AC [14, 137].

Cholinergic motor nerve terminals of the rat diaphragm also contain the vasoactive intestinal peptide (VIP) [138]. Neurochemical and electrophysiological data show that VIP acts presynaptically to facilitate ACh release at amphibian and mammalian NMJs [139]. Interestingly, VIP-induced facilitation of ACh release was only apparent when high extracellular levels of ADO accumulated at the neuromuscular synapse as a consequence of high-frequency neuronal firing episodes [46, 132]. Like that occurring with CGRP (see above), our findings show that synergism with A_2A_Rs is also required to trigger VIP-induced facilitation of ACh release from motor nerve terminals [14, 132].

Moreover, A_2A_Rs also play a pivotal role in the neuromuscular transmission facilitation caused by the neurotrophin brain-derived neurotrophic factor (BDNF). The source of BDNF at the skeletal motor endplate is still a matter of debate, even though it may be released from thrombin-activated skeletal muscle fibres [130]. Pre- and postsynaptic tropomyosin receptor kinase B (TrkB) receptors can mediate the actions of BDNF at the NMJ [130, 131, 140, 141]. BDNF-induced potentiation of synaptic transmission associated with increases in the release probability involves the functional coupling between A_2A_R-dependent PKA and neurotrophin-triggered PLCγ and mitogen-activated protein kinase pathway [130, 131]. Overall, these findings suggest a common pattern indicating that the neuromuscular influence of neuropeptides and/or neurotrophins critically depends on endogenous ADO generation to extracellular levels high enough to activate A_2A_R.

## Perisynaptic Schwann Cells (PSCs): a Third-party Player in Neuromuscular Transmission Tuning

### PSCs Regulate Neuromuscular Development and Homeostasis

PSCs play significant roles in NMJ synaptogenesis, development, and repair [2]. Since PSCs directly shield the NMJ, their importance for the development and maintenance of this specialised synapse has been extensively investigated over the years. At amphibian NMJs, PSCs appear shortly after the formation of the first few muscle connections and maintain close contact with the neuromuscular synapse during its development. Throughout ontogeny, PSCs extend sprouts that spread well beyond the borders of the NMJ, which lead to nerve terminal growth; these processes disappear at later stages to give rise to the mature NMJ architecture. As such, PSC sprouts likely to play an important role in synaptic growth and maturation at the developing NMJ [142]. In line with such evidence, selective ablation of PSCs from amphibian NMJs causes early (within a few days) retraction of nerve terminals, synaptic growth inhibition, and nerve-evoked muscle contraction impairment [143]. Regeneration of NMJs also implicates PSCs via mechanisms similar to those described for synaptogenesis. Following nerve injury, PSCs elaborate extensive sprouts to guide adequately the regenerating nerve terminals [144, 145].

Overall, PSCs account for important processes related to the formation, maintenance, and repair of the NMJ. By receiving inputs from both skeletal muscle fibres and motor neurons, PSCs may respond concurrently by expressing a plethora of molecular effectors. The relevance of PSCs in this process is highlighted given that their absence, or of its molecular effectors, significantly impairs the formation, maintenance and repair of NMJs, resulting in structural and functional abnormalities of motor endplates. Therefore, PSCs are situated to sense the functional state of motor endplates, which might be critical for the development and preservation of NMJ integrity [2, 146].

### PSCs Modulate the Neuromuscular Synaptic Transmission

In addition to their role in synaptogenesis, development, and repair, PSCs are crucial players in neurotransmission regulation both in amphibian [12, 13] and in mammalian [8–10] NMJs. This agrees with the tripartite arrangement concept for the neuromuscular synapse and complies with that found in several synapses of the CNS [147]. At the frog NMJ, injection of guanosine 5′-O-(3-thiotriphosphate) (GTPγS, a non-hydrolysable analogue of guanosine 5′-triphosphate) in PSCs reduces nerve-evoked transmitter release through pertussis-toxin-sensitive and insensitive G-proteins [13]. This suggests that nonspecific activation of G proteins in PSCs depresses neuromuscular transmission, with cholinergic and/or purinergic metabotropic receptors as strong candidates to fulfil this task. Contrariwise, Ca^2+^ mobilisation from IP_3_-sensitive stores in PSCs potentiates synaptic activity, while injection of the fast Ca^2+^ chelator, BAPTA, into these cells favours synaptic depression [12]. Overall, it has been proposed that mammalian PSCs may be important to decode, either facilitating or inhibiting synaptic activity based on intracellular Ca^2+^ oscillations and the dynamic interplay with purinoceptors [10]. Increasing evidence demonstrates a direct link between the activation of α7 nAChRs at the surface of PSCs and depression of the neuromuscular transmission [8, 9]. PSCs are also endowed with plasma membrane-bound purinoceptors, which may be critical for these cells to sense and adapt neuromuscular transmission on a moment-to-moment basis [9, 11]. Unravelling the complex network of interactions between different cells at the NMJ will certainly upgrade our understanding of the molecular mechanisms involved in the regulation of ACh release and the organisation of tripartite synapses, both in health and disease conditions.

### PSCs Modulate Synaptic Activity via Purinergic Signalling

The presence of purinoceptors, side by side with cholinoceptors and receptors for other signalling molecules [9, 11], allows PSCs to sense and regulate the neuromuscular synaptic activity through a variety of different mechanisms [8–10, 36]. ATP and ADO have been identified as putative gliotransmitters released from PSCs in response to synaptic activity to either inhibit or facilitate ACh release through the activation of various purinoceptor subtypes [8, 10].

**Fig. 2:**
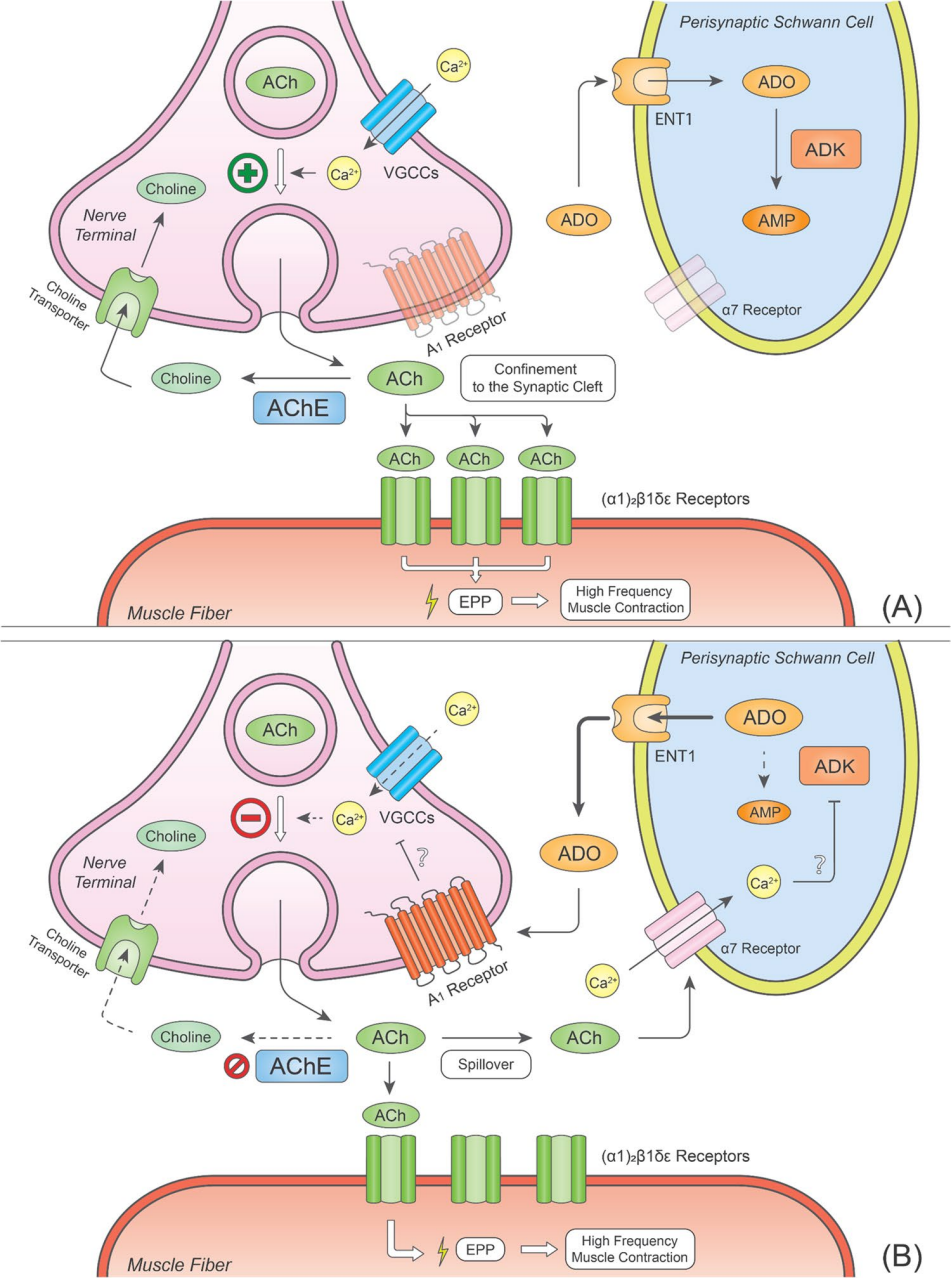
Sensing role PSCs to control ACh spillover from the neuromuscular synapse in the presence of cholinesterase inhibitors: on the role of α7 nAChRs-induced adenosine release and retrograde activation of A_1_ receptors. See text for details. Bold arrows indicate an increase in the strength of the proposed pathways; dashed arrows indicate a decrease in the strength of the proposed pathways. Predominant receptors operating in each condition appear as fully opaque, while non-predominant receptors appear as semi-transparent. The plus green symbol in panel (A) stands for enhancement of Ca^2+^ influx and acetylcholine release, while the minus red symbol in panel (**B**) accounts for a decrease in both Ca^2+^ influx and neurotransmitter release. Abbreviations: ACh, acetylcholine; AChE, acetylcholinesterase; ADK, adenosine kinase; ADO, ADO; AMP, adenosine 5′-monophosphate; ENT1, equilibrative nucleoside transporter 1; EPP, endplate potential; VGCC, voltage-gated Ca^2+^ channel

The presence of functional homo-pentameric nAChRs containing α7 subunits (α7 nAChRs) was demonstrated on the surface of PSCs from adult rodents by immunohistochemistry [8, 9]. These receptors were initially assumed to be located on presynaptic nerve terminals, most likely because of their ability to affect electrophysiological recordings using sharp intracellular microelectrodes impaling skeletal muscle fibres, which do not distinguish presynaptic from perisynaptic-originated signals [148]. α7 nAChRs were also identified in the sarcolemma of developing or denervated NMJs [9, 149], but they were absent from motor nerve terminals and skeletal muscle fibres of healthy adult animals [9, 150]. Unlike the muscle-type α1-containing nAChRs, the α7 nAChRs desensitise rapidly, exhibit higher Ca^2^ permeability, and are equally activated by ACh and its metabolite, choline [8, 151]. The perisynaptic localisation of these receptors on PSCs prompts a sensing activity that is predominant when ACh spills over from the motor endplate during intense neuronal firing or in the presence of cholinesterase inhibitors used to reverse surgical neuromuscular block and myasthenic crisis. α7 nAChRs present at the surface of PSCs operate a negative-feedback mechanism to downregulate nerve-evoked ACh release (Fig. 2) [9].

The fact that (1) α7 nAChRs are absent from motor nerve terminals and that (2) α7 nAChR-induced inhibition of transmitter exocytosis required inhibition of butyrylcholinesterase anchored to the surface of PSCs raised questions about (i) the physiological meaning of the need for surplus ACh accumulation at the neuromuscular synapse and (ii) the chemical nature of the gliotransmitter responsible for the communication between the PSC and the underlying motor nerve terminal (Fig. 2a). Using neurochemical and real-time fluorescence microscopy assays, our group showed that selective activation of α7 nAChRs increased intracellular Ca^2+^ inside PSCs while decreasing transmitter exocytosis elicited by high-frequency nerve stimulation bursts [8]. All these effects were abrogated by the glial cell metabolic uncoupler, sodium fluoroacetate [152, 153]. Data also showed that α7 nAChRs control ACh spillover from the neuromuscular synapse by promoting ADO outflow from PSCs via ENT1, with subsequent retrograde activation of presynaptic inhibitory A_1_Rs. These findings demonstrate that ADO is the gliotransmitter involved in this mechanism. This was concluded because (1) removal of endogenous ADO with ADA, (2) inhibition of ADO release via ENT1 transporter, and (3) blockage of presynaptic A_1_Rs, all prevented nerve-evoked ACh release inhibition caused by α7 nAChR activation on PSCs [8]. Moreover, the pharmacology of α7 nAChR-induced down-modulation of ACh release was remarkably similar to that observed by inhibiting ADK [154], a situation that is known to increase the intracellular accumulation of the nucleoside, thus forcing its translocation to the extracellular milieu via ENTs (Fig. 2b).

There is, however, a gap in our knowledge about the molecular mechanism linking α7 nAChR activation to ADO outflow from PSCs via ENT1. It is known that both human and mouse ENT1 are directly phosphorylated by PKA and PKC [155] and that PKC-mediated phosphorylation of ENT1 increases its transport efficacy and/or mobilisation to the plasma membrane, thus contributing to facilitating ADO outflow [156, 157]. Alternatively, PKC-mediated ADK inhibition may foster ADO outflow via ENT1 by increasing the intracellular concentration of the nucleoside [154]. Experimental data ruled out the involvement of PLC and IP_3_ receptor-mediated mechanisms [8], as well as the participation of atypical PKC isoforms [158] in the inhibitory role of α7 nAChR on neuromuscular transmission. This suggests that other effectors, like Ca^2+^/calmodulin-dependent protein kinase II and ryanodine receptors, may be involved instead [159].

Nerve-evoked PSC Ca^2+^ transients increase in magnitude and duration when high-frequency tetanic trains are delivered in the presence of the cholinesterase inhibitor, neostigmine [8]. These findings may be clinically relevant given that cholinesterase inhibitors are frequently used to improve neuromuscular transmission in patients with *myasthenia gravis* and to reverse the residual neuromuscular block caused by non-depolarising muscle relaxants. Activation of perisynaptic α7 nAChRs by ACh spilling over from the NMJ may also explain the paradoxical reduction of nerve-evoked neurotransmitter release (e.g. “train-of-four fade”, meaning the reduction of the fourth to the first twitch height in a stimulation train delivered at 2 Hz frequency) observed with widely used cholinesterase inhibitors, like neostigmine, leading to partial or longer recovery from the neuromuscular block even in the presence of atropine [160–162]. A similar mechanism may occur regarding the effects of muscle relaxants exhibiting significant cholinesterase activity, like cis-atracurium [163–165].

**Fig. 3:**
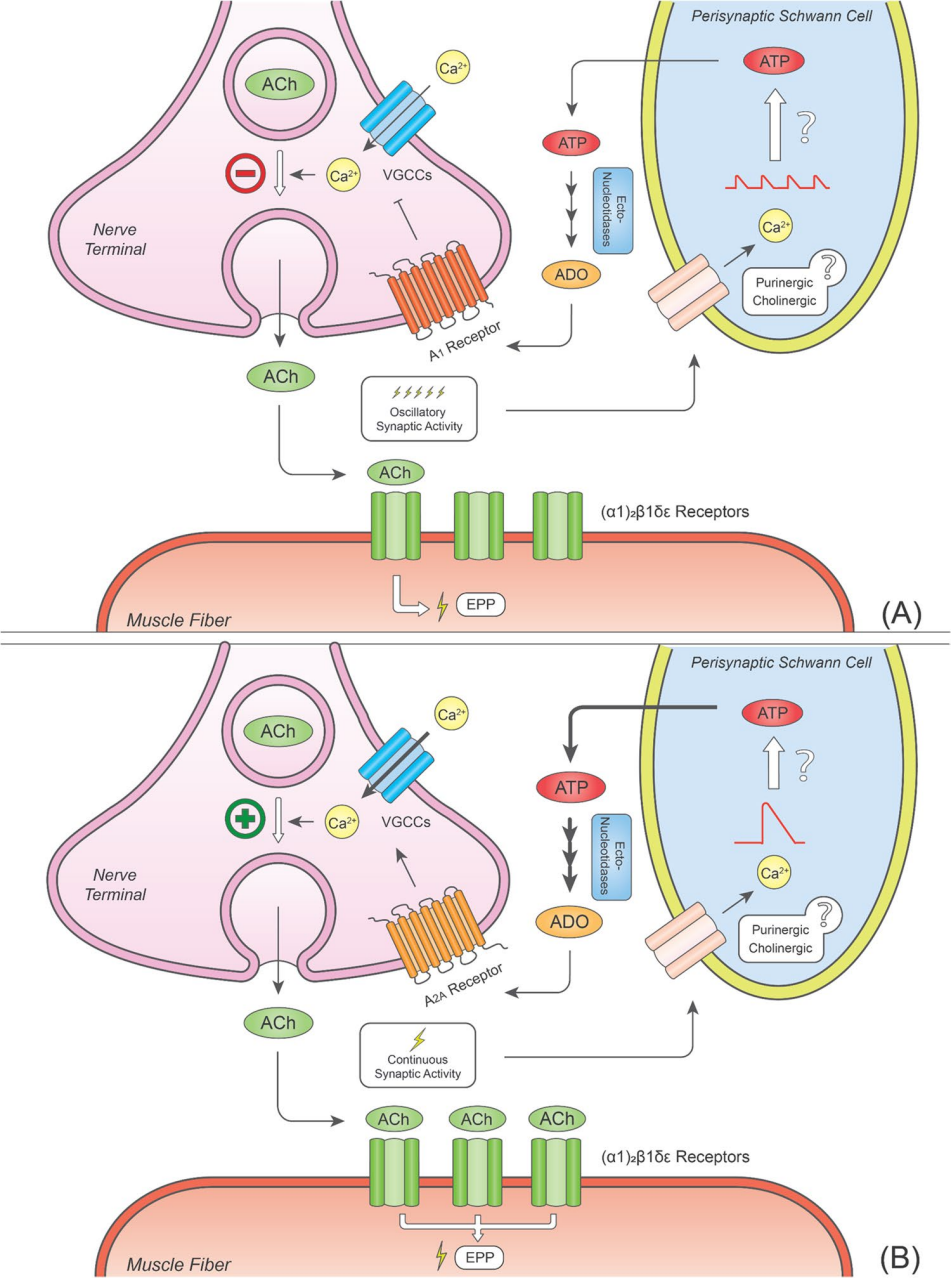
PSCs detect patterns of synaptic activity and subsequentially provide feedback to motor neurons by releasing ATP. Depending on the paradigm of neuronal firing, ATP released by PSCs can either decrease (**A**) or increase (**B**) ACh release via its rapid hydrolysis into adenosine and activation of A_1_ or A_2A_Rs, respectively. See text for details. Bold arrows indicate an increase in the strength of the proposed pathways. The minus red symbol in panel (A) stands for a decrease in Ca^2+^ influx and ACh release, while the plus green symbol in panel (B) accounts for an increase in both Ca^2+^ influx and neurotransmitter release. Abbreviations: ACh, acetylcholine; ADO, adenosine; ATP, adenosine 5′-triphosphate; EPP, endplate potential; VGCC, voltage-gated Ca^2+^ channel

The nerve stimulation pattern also differentially affects synaptic activity at the mouse NMJ, and these nuances are intimately linked to the glial modulation of neurotransmitter release (Fig. 3). At the mouse soleus, prolonged continuous stimulation trains have been shown to favour post-tetanic potentiation and phasic Ca^2+^ responses in PSCs, while intermittent bursting paradigms favour post-tetanic depression and oscillatory Ca^2+^ responses in these cells [10, 36]. Chelation of Ca^2+^ transients inside glial cells abolishes these effects, strengthening the idea that PSCs interpret and effectively modulate synaptic activity using Ca^2+^ as a second messenger [12, 13]. By mimicking Ca^2+^ oscillations inside PSCs using flash photolysis of caged Ca^2+^, Todd and co-workers [10] showed that (i) synaptic plasticity events were prevented by inhibiting the extracellular ADO formation from the catabolism of adenine nucleotides, and that (ii) the post-tetanic depression and potentiation were dependent on the activation of A_1_R (Fig. 3a) and A_2A_R (Fig. 3b), respectively. These findings suggest that PSCs release ATP in response to Ca^2+^ transients secondary to motor neuron activation which after being sequentially metabolised into ADO activates A_1_R or A_2A_R depending on the stimulation paradigm [46].

Despite the aforementioned findings, it is still unclear which receptors are involved in the ability of PSCs to detect and modulate the activity of the neuromuscular synapse. The broad-spectrum nicotinic antagonist, *d*-tubocurarine, is commonly used to paralyse skeletal muscle fibres during electrophysiological recordings, which might eliminate any source of modulation undertaken by α7 nAChRs located in the plasma membrane of PSCs. It is likely that under such conditions PSCs detect and modulate nerve-evoked ACh release via muscarinic and/or purinergic receptors [11], resulting in ATP and/or ADO release to the synaptic cleft. The presence of mAChRs has been functionally demonstrated in PSCs of amphibian NMJs; this assumption was based on the fact that both electrical nerve stimulation and exogenous application of mAChR agonists were able to induce PSCs Ca^2+^ oscillations in the presence of *d*-tubocurarine [166, 167]. This conclusion is strengthened by the fact that both pertussis-toxin-sensitive and insensitive G-proteins and Ca^2+^ recruitment form PSC internal stores were able to influence neuromuscular transmission at the frog NMJ [12, 13]. In this context, it might be possible that the effects attributed to facilitatory M_1_Rs at motor endplates reflect, at least in part, the effect of a retrograde gliotransmitter released by PSCs [168]. Likewise, activation of P2X, P2Y, and A_1_Rs on PSCs causes intracellular Ca^2+^ oscillations in these cells [11, 34], strengthening the neuromuscular plasticity phenomena reported by Todd and co-workers [10]. However, there are still gaps in our knowledge concerning the putative role of perisynaptic muscarinic and purinergic receptors on neuromuscular transmission adaptations, which are worth pursuing in the future.

Considering that A_1_R may be present and operate Ca^2+^ rises inside PSCs at mammalian NMJs [11], one may hypothesise that these receptors act synergistically with the α7 nAChR-mediated sensing mechanism to control spillover of the neurotransmitter from the motor endplate [8, 9]. Thus, α7 nAChR-induced Ca^2+^ oscillations may be further strengthened by fostering ADO outflow from PSCs, which results in autocrine activation of A_1_Rs. While this mechanism may be functionally relevant to amplify the sensing role of the α7 nAChR on PSCs, it still does not explain the inhibitory repercussion of this receptor on neurotransmitter exocytosis, unless ADO simultaneously acts as a retrograde gliotransmitter via inhibitory A_1_Rs on motor nerve terminals [8, 46, 78]. Compelling experimental data indicate that the inhibitory tone operated via A_1_Rs on nerve-evoked ACh release is normally silent during high-frequency neuronal bursts [46] unless α7 nAChR on PSCs are activated to restrain ACh spillover from the neuromuscular synapse [8]. Thus, the integrated action of α7 nAChR and A_1_Rs on PSCs may act as a secondary self-sustained ADO-mediated break supplementing the muscarinic M_2_R auto-inhibition to avoid exhaustion of ACh reservoirs during prolonged high-frequency neuronal activity, which would endanger the integrity of motor nerve terminals and, thus, neuromuscular transmission efficacy.

## Concluding Remarks

At mature NMJs, adenine nucleotides are intimately involved in the control of ACh release from stimulated motor nerve terminals, as well as in muscular contractile activity. ATP is released together with ACh from activated nerve terminals. The release of ATP from contracting skeletal myotubes has also been observed. While the latter may involve pannexin-1 hemichannels, ATP releases together with ACh implies exocytosis of synaptic vesicles. Once in the extracellular space, ATP may be rapidly broken down into ADP, AMP, and ADO [24, 60]. However, there are still gaps in our knowledge concerning the mechanisms implicated in the control of neuromuscular transmission by adenine nucleotides, though data suggest that they might differ between species and developing or adult motor endplates.

ADP-sensitive P2Y_12_ and P2Y_13_Rs play inhibitory roles in quantal ACh release from nerve terminals of amphibian and mammalian NMJs, respectively [41, 59]. *G*_q/11_-coupled P2YRs were also involved in the inhibition of non-quantal ACh release [39, 40], yet their full molecular and pharmacological characterisation is still missing. Likewise, activation of P2YRs on PSCs triggers intracellular Ca^2+^ transients, but the physiological meaning of this effect remains to be elucidated [34]. Regarding ATP-sensitive P2XRs, evidence has been gathered indicating that they might be present on presynaptic motor nerve terminals as well as on PSCs. Activation of neuronal P2X7Rs is associated with increases in vesicular exocytosis, while activation of P2XRs on PSCs favours intracellular Ca^2+^ oscillations [34, 49]. Given the limited information regarding P2 receptor characterisation and function at the neuromuscular junction, further studies are encouraged before any firm conclusion can be drawn from their role in neuromuscular transmission and plasticity.

At most motor endplates, ADO can be released as such or originated from the extracellular catabolism of release ATP. The nucleoside plays a predominant inhibitory role in nerve-evoked ACh release under resting conditions via the activation of inhibitory A_1_Rs [37, 38, 46, 88]. This scenario dramatically changes during high-frequency stimuli or upon focal depolarisation of motor nerve terminals, where co-localised A_2A_Rs contribute to facilitating neurotransmitter release [46, 86, 111], which may be critical to overcome the tetanic depression of the neuromuscular transmission (Fig. 1a and 1b) [77]. Low-affinity A_2B_ and A_3_Rs have also been identified at the mouse NMJ; the presence of the A_3_R subtype raises the possibility that the ADO metabolite inosine can also inhibit nerve-evoked ACh release [104, 108].

Close association and putative functional interplay between the ADO-generating enzyme, ecto-5′-nucleotidase/CD73, and facilitatory A_2A_Rs has been demonstrated. This explains why the extracellular breakdown of adenine nucleotides delivers the nucleoside directly to this receptor subtype, thus facilitating ACh release in many different synapses [24, 95–98]. Activation of A_2A_Rs fosters Ca^2+^ influx into stimulated motor nerve terminals through the recruitment of normally “quiescent” L-type (Ca_v_1) channels by an AC/cAMP/PKA-dependent pathway; this mechanism contributes to bypass P/Q-type (Ca_v_2.1) channel desensitisation and sustains ACh release during high-frequency neuronal bursts [46, 75, 77, 86]. Failure of this mechanism has been associated with neuromuscular transmission deficits in myasthenic patients, leading us to hypothesise that targeting the endogenous ADO formation by ecto-5′-nucleotidase/CD73 and A_2A_R activation might restore neuromuscular competence while also suppressing activation of the immune system in *myasthenia gravis* patients [28, 69, 114].

ADO also plays a pivotal role in regulating the cholinergic and peptidergic modulation of ACh release at the NMJ. Activation of A_1_Rs suppresses cholinergic auto-inhibition by M_2_Rs, thus allowing the M_1_R-mediated facilitation to play a predominant role in resting conditions [6, 73]. As a shift from A_1_-inhibitory to A_2A_-facilitatory tonus occurs during high neuronal firing rates, the activity of facilitatory M_1_Rs and α3β2 nAChRs is suppressed, and that of M_2_Rs is unmasked [6, 121, 123]. Furthermore, activation of A_2A_Rs by ADO also unmasks the facilitatory role of neuropeptides such as CGRP [14], VIP [132], and BDNF [130, 131] by a mechanism involving the AC/cAMP/PKA pathway.

As emphasised above, PSCs are endowed with A_1_Rs coupled to intracellular Ca^2+^ mobilisation [11, 34, 104]. Autocrine stimulation of A_1_Rs by ADO released from PSCs (via ENT1 transporters) in response to α7 nAChR activation may significantly enhance the sensing ability of the latter receptors to control ACh spillover from the motor endplate region and prevent exhaustion of the neurotransmitter during high-frequency neuronal bursts; this could otherwise endanger subsequent neuromuscular transmission performance and motor endplate integrity [8, 9]. Thus, A_1_Rs located on PSCs and motor nerve terminals may act in tandem to control neurotransmitter exocytosis once α7 nAChRs sense an excess of ACh emerging from the neuromuscular synapse and trigger the release of ADO, which most likely functions as a retrograde gliotransmitter at mammalian motor endplates. This newly evidenced mechanism may add an explanation to the paradoxical reductions of nerve-evoked neurotransmitter release often observed in medical interventions with cholinesterase inhibitors, like neostigmine [160–162], and with skeletal muscle relaxants exhibiting significant cholinesterase activity, like cis-atracurium [163–165].

In summary, purines play extremely important and dynamic roles in modulating neuromuscular transmission in health and disease conditions. Adenine nucleotides and nucleosides are also engaged in the mechanisms by which PSCs govern neuromuscular transmission, emphasising the role of these purines as gliotransmitters in tripartite synapses, like the NMJ. Using animal models of neuromuscular diseases, it became evident that fostering the A_2A_R-mediated reinforcement of the neuromuscular transmission may be a good therapeutic strategy to overcome the neuromuscular deficits associated with myasthenia gravis and ALS, providing the conclusion of ongoing preclinical studies and the development of novel and safer drug compounds entitled to be used in clinical trials.

## Data Availability

Not applicable.
